# Latest advances in nanodrug delivery systems for modulating the immune microenvironment in triple-negative breast cancer

**DOI:** 10.3389/fimmu.2026.1793737

**Published:** 2026-07-17

**Authors:** Xiaoya Dong, Fengxin Cui, Lei Wang, Mai Wu, Yiling Liu, Lehan Gao, Junyao Xie, Xinru Lin, Jianming Sun, Xiaorong Zhou, Run Meng

**Affiliations:** 1 Department of Immunology, Medical School, Nantong University, Nantong, China; 2 Department of Andrology, Seventh People's Hospital of Shanghai University of Traditional Chinese Medicine, Shanghai, China; 3 The First School of Clinical Medicine, Nanjing Medical University, Nanjing, China

**Keywords:** drug delivery, EPR effect, nanocarrier, triple-negative breast cancer, tumor immune microenvironment

## Abstract

The tumor immune microenvironment (TIME), composed of tumor cells, immune/stromal cells, cytokines, and other components, plays a central role in determining tumor immunogenicity and response to therapy. The balance between effector T/NK cells and immunosuppressive populations such as regulatory T cells (Tregs), myeloid-derived suppressor cells (MDSCs), and M2-like tumor-associated macrophages (TAMs) determines whether tumors remain “cold” or become “hot”. Triple-negative breast cancer (TNBC) remains challenging to treat because it lacks estrogen receptor (ER), progesterone receptor (PR), and human epidermal growth factor receptor 2 (HER2) targets and exhibits high heterogeneity. To address these limitations, tumor microenvironment (TME)-targeted nanocarriers have emerged as a promising strategy. By exploiting features such as hypoxia, acidity, redox imbalance, and abnormal vascular and mechanical cues, these systems enable prolonged circulation, active targeting, and stimulus-responsive release, thereby enhancing the efficacy of therapies such as immune checkpoint blockade. This review summarizes major nanoplatforms and therapeutic strategies, while highlighting translational barriers including TIME heterogeneity, enhanced permeability and retention (EPR) effect, and protein corona formation. Finally, this review explains why patient stratification should be incorporated into the future development of TNBC nano-immunotherapy and argues for simplified, reproducible nanocarrier designs to support clinically applicable precision treatment.

## 1 Introduction

### 1.1 Concept of the tumor immune microenvironment and its relevance to TNBC

TIME refers to a complex ecosystem within the tumor site composed of tumor cells, immune cells, fibroblasts, vascular endothelial cells, as well as cytokines, chemokines, exosomes, and the extracellular matrix, representing the immunologically relevant components of the broader tumor microenvironment that is directly associated with immune responses (1). As a core constituent of the tumor microenvironment, TIME is not a simple assemblage of cells and molecules. Rather, it comprises highly heterogeneous functional units formed through intricate signal-network interactions among its components (2). Within this system, effector T cells, B cells, NK cells, and dendritic cells exert antitumor functions, whereas Tregs, MDSCs, and M2-type TAMs, among others, collectively sustain an immunosuppressive environment that facilitates tumor-cell immune evasion (3). Beyond immune cells, cancer-associated fibroblasts can remodel the TIME architecture by secreting extracellular matrix components and can concurrently recruit immunosuppressive cells through chemokine secretion (4). Vascular endothelial cells provide nutritional support for tumor cells and can restrict immune-cell infiltration. TIME is not static but is continuously reshaped during tumor initiation, progression, and treatment: tumor cells reprogram the microenvironment by secreting immunosuppressive factors, reconstructing vasculature, and altering metabolism and acid–base conditions. Meanwhile, distinct immune-cell populations can either suppress or promote tumor-cell proliferation, invasion, and therapeutic sensitivity (5). Therefore, TIME is both a key component of tumor biology and an essential theoretical basis for understanding differences between “cold” and “hot” tumors and between treatment sensitivity and resistance. Among solid tumors, TNBC represents a particularly relevant setting for TIME-oriented research because its clinical aggressiveness is accompanied by marked immune heterogeneity (6). A subset of TNBC lesions contains abundant tumor-infiltrating lymphocytes and elevated immune checkpoint expression, whereas other lesions show immune exclusion or dominance of suppressive myeloid, regulatory T-cell, macrophage, stromal, and vascular programs (7). This coexistence of immune activation and immune restraint helps explain the variable response of TNBC to immunotherapy and supports the development of microenvironment-targeted therapeutic strategies (8, 9).

The conceptual foundation of TIME has evolved from the recognition that immune-cell composition and spatial organization within tumors are closely associated with clinical outcome. Galon et al. demonstrated that the type, density, and location of intratumoral immune cells could predict patient prognosis, establishing the basis for the concept of “immune contexture” (10, 11). This framework was later extended by Chen and Mellman, who proposed the “cancer–immunity set point” and classified tumor immune phenotypes into inflamed, immune-excluded, and immune-desert patterns (12). Subsequent reviews further standardized the term TIME and its major categories, providing a common conceptual framework for interpreting tumor immune heterogeneity and therapeutic response (13–15).

### 1.2 Significance and roles of regulating the tumor immune microenvironment

The central significance of regulating the tumor immune microenvironment lies in reshaping local immune homeostasis within tumors, reversing immunosuppression, and restoring antitumor immune function, thereby inhibiting tumor progression and improving therapeutic efficacy (**Table 1**). This role spans the entire course of tumorigenesis, tumor development, and treatment, making it particularly important in precision oncology. In terms of its regulatory significance and functional impact, the tumor immune microenvironment is involved in nearly every stage of tumor evolution, including initiation, clonal evolution, hematogenous or lymphatic metastasis, and responses to radiotherapy/chemotherapy, targeted therapy, and immunotherapy (28). In immunosuppressive TIME, effector T-cell exhaustion, impaired antigen presentation, and high expression of immune checkpoint molecules (e.g., PD-1/PD-L1 and CTLA-4) enable tumors to continue growing in an “immune-indifferent” state (29). By contrast, an inflamed TIME enriched with tumor-infiltrating lymphocytes (TILs) often indicates a more favorable prognosis and a higher response rate to immunotherapy (30). Modern cancer therapy is increasingly shifting from “directly killing tumor cells” to “concurrently remodeling the microenvironment,” using immune checkpoint inhibitors, CAR-T cells, cancer vaccines, oncolytic viruses, and drugs targeting the TME/TIME (e.g., agents against angiogenesis, metabolism, and cytokines) to relieve immunosuppression and enhance antitumor immunity, thereby achieving more durable and systemic therapeutic effects (31). In addition, regulating TIME can reduce metastatic risk. Studies have shown that vascular endothelial growth factor secreted by M2-type TAMs can promote tumor angiogenesis and lymphangiogenesis, whereas TAM-targeted regulatory strategies can inhibit tumor angiogenesis and reduce routes of dissemination (32). In clinical practice, TIME-related biomarkers, such as PD-L1 expression, TMB (tumor mutational burden), and the abundance, composition, and spatial distribution of tumor-infiltrating immune cells, have become important criteria for guiding immunotherapy selection, and deeper investigation of their regulatory mechanisms provides clear directions for developing novel anticancer therapeutics (33). Therefore, in TNBC, a systematic understanding of TIME and its strategic modulation may provide an important basis for improving therapeutic efficacy and extending patient survival (34). This therapeutic relevance is particularly evident in TNBC, where immune activation does not always translate into effective tumor rejection. Tumors with abundant lymphocytic infiltration and checkpoint expression may be more sensitive to immune checkpoint blockade, whereas immune-excluded lesions with dysfunctional T cells, suppressive myeloid cells, regulatory T cells, tumor-associated macrophages, and abnormal stromal or vascular barriers remain less responsive. Accordingly, TIME modulation should be viewed as a strategy to restore productive immune engagement, improve immune-cell access and persistence, and strengthen the efficacy of chemotherapy, immune checkpoint blockade, and other combination therapies (35).

**Table 1:** Significance and effects of different regulatory targets.

Regulatory target	Significance of regulation	Effects	References
CD8^+^ effector T cells/Th1	Strengthening the main antitumor immune effector arm	Promoting tumor cell killing, secreting IFN-γ, and establishing immunological memory	(16, 17)
Tregs	Suppressing antitumor immunity	Suppressing effector T cells, promoting immune evasion, and contributing to therapeutic resistance	(18, 19)
MDSCs	Targeting or reprogramming the immunosuppressive myeloid compartment	Reducing MDSC-mediated immunosuppression, limiting recruitment or polarization of suppressive immune cells, and thereby inhibiting tumor progression, invasion, and metastasis	(20, 21)
TAM (M2 → M1)	Shifting from pro-tumor to anti-tumor activity	Increasing phagocytosis and antigen presentation; and promoting vascular normalization	(22, 23)
DCs and antigen presentation	Enhancing priming and cross-presentation	Upregulating co-stimulatory signals and improving primary T-cell priming	(24, 25)
Immune checkpoints (PD-1/PD-L1, CTLA-4, etc.)	Blocking inhibitory immune checkpoint pathways	Reversing T-cell exhaustion, restoring effector T-cell function, and promoting clonal expansion of tumor-reactive T cells	(26, 27)

The rationale for TIME modulation emerged from the broader development of tumor microenvironment research, cancer immunoediting, and immune checkpoint biology. Pivotal theoretical and clinical contributions by Schreiber (36), Hanahan and Weinberg (37), Chen and Mellman (38), Allison (39, 40), Honjo (41, 42) and others established that durable cancer control requires both tumor-cell targeting and relief of immune suppression. In TNBC, this framework provides a mechanistic basis for integrating chemotherapy, immunotherapy, and microenvironment-directed nanomedicine.

### 1.3 Characteristics of triple-negative breast cancer

TNBC refers to a subtype of breast cancer that is negative for ER, PR, and HER2, accounting for approximately 10%–20% of all breast cancers (43). Because these canonical therapeutic targets are absent, TNBC management relies heavily on chemotherapy, immune checkpoint blockade in selected patients, PARP inhibitors in BRCA-mutated disease, antibody-drug conjugates, and rational combination strategies rather than endocrine or HER2-directed therapy. Clinically, TNBC occurs more frequently in younger women (particularly those <40 years of age) and is closely associated with BRCA1/2 mutations and a family history of hereditary susceptibility. These tumors often present with larger masses, high histologic grade (commonly grade III), and a high mitotic index, and may develop lymph-node or distant metastases at an early stage. Common metastatic sites include the lung, liver, brain, and bone, with TNBC showing a particular tendency toward visceral and brain metastases. Metastatic TNBC is generally associated with poor survival outcomes, with reported median overall survival commonly ranging from approximately 8 to 13 months in real-world cohorts, although survival varies according to metastatic site, disease burden, and treatment availability (44, 45). Meanwhile, TNBC is highly heterogeneous and can be stratified by molecular subtyping into basal-like, immune-enriched, mesenchymal, and other subtypes, which exhibit marked differences in mutational landscapes, patterns of signaling-pathway activation, and interactions with the TIME (46). In addition to immune-related heterogeneity, TNBC also exhibits marked metabolic and receptor-level alterations that can be exploited for targeted nanomedicine. Transferrin receptor 1 (TfR1, also known as CD71), encoded by TFRC, is a type II transmembrane glycoprotein responsible for binding transferrin-bound iron and mediating cellular iron uptake through receptor-mediated endocytosis (47). Because rapidly proliferating tumor cells require increased iron for DNA synthesis, mitochondrial metabolism, and cell-cycle progression, TfR1 is frequently upregulated in malignant tumors and has been widely explored as a tumor-targeting receptor (48). In TNBC, the relevance of TfR1 lies not only in its association with iron-metabolic reprogramming and aggressive tumor growth, but also in its potential to facilitate receptor-mediated internalization of transferrin-, antibody-, peptide-, or H-ferritin-based nanocarriers. Therefore, TfR1 provides an important mechanistic basis for the later use of TfR1-targeted nanoformulations to improve tumor localization, intracellular uptake, and therapeutic delivery efficiency in TNBC (49). From the perspective of the immune microenvironment, TNBC displays distinctive TIME phenotypes. On the one hand, TNBC often has a relatively high tumor mutational burden, generating more tumor neoantigens and facilitating immune recognition. Consequently, a subset of TNBC constitutes “hot tumors” with substantial infiltration of CD8^+^ T cells and dendritic cells, and patients with such tumors show higher response rates to immune checkpoint inhibitors. On the other hand, some TNBCs retain pronounced immunosuppressive features, such as enrichment of M2-type tumor-associated macrophages and Treg cells that secrete large amounts of inhibitory cytokines (e.g., IL-10 and TGF-β), or high expression of immune checkpoint molecules, leading to functional exhaustion of effector immune cells. Notably, a considerable proportion of TNBC exhibits high levels of tumor-infiltrating lymphocytes and elevated PD-L1 expression (50). This “immunologically active yet suppressed” profile provides a biological rationale for the use of immune checkpoint inhibitors and antibody–drug conjugates and has demonstrated potential to improve progression-free survival and overall survival in recent clinical trials. From a therapeutic perspective, TNBC is also characterized by a relatively limited therapeutic window (51). Because ER, PR, and HER2 are absent, treatment cannot rely on endocrine or HER2-directed strategies and instead depends largely on chemotherapy, immune checkpoint blockade in selected patients, PARP inhibitors in BRCA-mutated disease, antibody-drug conjugates, and rational combinations (52). However, dose intensification is often constrained by systemic toxicity, while insufficient drug exposure may fail to control rapidly progressive or recurrent disease (53). This narrow balance between antitumor efficacy and treatment-related toxicity is particularly relevant for nanomedicine design, because tumor-targeted accumulation and stimulus-responsive release may increase local therapeutic exposure while reducing nonspecific damage to normal tissues (54).

Contemporary TNBC management has evolved through accumulated clinical evidence and now includes chemotherapy, PARP inhibitors for BRCA-mutated disease, immune checkpoint blockade in selected patients, antibody-drug conjugates, and rational combination strategies. Chemotherapy remains a major backbone of treatment, while PARP inhibitors, PD-1/PD-L1 blockade, and antibody-drug conjugates have expanded the therapeutic landscape for molecularly or immunologically defined patient subsets (55–58). These advances provide the clinical context for developing nanocarrier-based strategies that may improve drug delivery, reduce systemic toxicity, and enhance immune modulation in TNBC.

### 1.4 Potential value of modulating the immune microenvironment in TNBC

Modulating the immune microenvironment in triple-negative breast cancer has substantial potential clinical value. On the one hand, the high mutational and neoantigen burdens of TNBC provide targets for immune recognition, yet pervasive factors within its TIME, such as T-cell exhaustion, infiltration of immunosuppressive myeloid populations, and immunometabolic reprogramming, constrain spontaneous antitumor immunity (59). Strategies including blockade of immune checkpoints (e.g., PD-1/PD-L1 and CTLA-4) to restore T-cell function, combination with chemotherapy, radiotherapy, or antibody–drug conjugates to induce immunogenic cell death and enhance antigen release and presentation, and targeted interventions against tumor-associated macrophages, MDSCs, and aberrant vasculature provide a potential strategy to remodel the immunosuppressive TIME and sensitize “immune-cold” TNBC to immunotherapy (60). On the other hand, with advances in single-cell omics and spatial transcriptomics, refined stratification of the TNBC immune microenvironment will facilitate the development of risk-assessment models and treatment decision systems based on TIME features, enabling truly precise immunotherapy and optimization of combination-regimen strategies (61). Moreover, modulating the TNBC tumor immune microenvironment may reduce the risk of recurrence and metastasis and improve long-term outcomes (62). TNBC recurrence frequently occurs within three years after treatment, and relapse is closely linked to an immunosuppressive state within the TIME. Emerging evidence suggests that postoperative immune modulation may help reduce recurrence risk, although direct clinical validation in TNBC remains limited (63). Notably, with the development of gene-editing technologies, the efficacy of CAR-T cell therapy in TNBC also depends on TIME modulation, and engineering CAR-T cells to resist immunosuppressive signals within the TIME can enhance their survival and cytotoxic activity in tumor tissues (64).

In summary, the tumor immune microenvironment is not only a key window for understanding tumor biology and heterogeneity in therapeutic responses, but also a crucial avenue for future innovation in TNBC treatment strategies and prognostic improvement, with far-reaching implications for clinical translation and individualized therapy.

### 1.5 Scope and unique contribution of this review

Existing reviews have generally focused either on TNBC immunotherapy, on nanocarrier formulation, or on broad cancer nanomedicine. The present review is positioned at the intersection of these fields. Compared with previous reviews that separately discuss TNBC immunotherapy or nanocarrier-based delivery, this review integrates TIME heterogeneity, nanocarrier design logic, immune-remodeling mechanisms, and translational bottlenecks into a unified framework for precision TNBC nano-immunotherapy.

Accordingly, the review is organized to move from biological rationale to material design, mechanistic immune modulation, comparative platform evaluation, clinical-trial evidence, translational barriers, and multi-omics-guided precision nanotherapy. This structure is intended to provide not only a descriptive overview but also a critical framework for evaluating which nanocarrier strategies are most likely to become clinically actionable in TNBC.

## 2 Types of nanocarriers and their construction methods

### 2.1 Protein-based nanocarriers

Nanocarriers are widely used in cancer therapy to improve drug stability, tumor-site delivery, co-delivery capacity, and systemic safety. In TNBC, where aggressive progression, limited therapeutic targets, and drug resistance remain major challenges, nanocarriers provide an important strategy for improving chemotherapy, gene therapy, immunotherapy, and combination treatment (65, 66).

Protein-based nanocarriers, constructed from natural or recombinant proteins, offer favorable biocompatibility, biodegradability, and chemical modifiability (67). In TNBC, they mainly include albumin-based systems, structural protein-based particles, self-assembling protein nanocages, virus-like particles (VLPs), and other functional protein nanoparticles. Albumin is the most clinically representative plasma protein carrier, with albumin-bound paclitaxel already used as a clinical albumin-based formulation (68). At the preclinical level, albumin-based platforms have been evaluated for delivering taxanes, anthracyclines, and nucleic acid therapeutics in TNBC-related cell and mouse tumor or metastasis models. For example, doxorubicin-carried albumin nanocages have been examined in aggressive breast cancer and lymph-node metastasis models, whereas albumin-binding siRNA conjugates have been tested in human cancer cells and mouse xenograft models (69, 70). Structural protein-based systems, such as silk fibroin-based local delivery scaffolds, are still mainly supported by *in vitro* and murine tumor evidence and should therefore be regarded as preclinical delivery platforms rather than established TNBC therapies (71–73).

Among self-assembling protein nanocages, ferritin is particularly representative because its hollow cavity enables drug loading, while subunit engineering supports targeted delivery and controlled release (74). In TNBC, H-ferritin nanocages loaded with doxorubicin have shown improved antitumor and antimetastatic effects compared with free doxorubicin in patient-derived xenograft and syngeneic mouse models (75). More recently, H-ferritin nanocages have also been used to deliver a CDK9-targeting PROTAC in TNBC, improving TfR1-mediated uptake, lysosomal pH-triggered release, CDK9 degradation, antitumor efficacy, and systemic safety (76).

VLPs represent another protein-based platform. Because they retain capsid-like self-assembled structures but lack viral genomes, they are suitable for ligand display and delivery of drugs, proteins, or nucleic acids. For example, GE11-modified HBc VLPs have been used to deliver doxorubicin to EGFR-overexpressing TNBC, and modular HBV VLPs can encapsulate therapeutic proteins for EGFR-directed delivery (77, 78). Other functional protein systems include lactoferrin-based hybrid nanodrugs, which have been used for the co-delivery of HDAC6 and LDH inhibitors in TNBC. By simultaneously affecting stress-related signaling and lactate metabolism, this strategy may promote metabolic reprogramming and immune sensitization (79).

From a formulation perspective, albumin- and silk fibroin-based particles are mainly prepared by desolvation/antisolvent precipitation, emulsification-solidification, or solvent-induced self-assembly, with drug loading usually occurring during protein aggregation and crosslinking (80, 81). In contrast, ferritin and VLPs rely more on post-expression self-assembly, pH-triggered disassembly-reassembly, or genetic fusion strategies for cargo encapsulation and surface functionalization (82). Therefore, the key challenge for protein-based nanocarriers in TNBC is not merely nanoparticle fabrication, but rational engineering to target receptors such as EGFR and TfR1, improve tumor localization and cellular uptake, and enhance the co-delivery efficiency of taxanes, doxorubicin, PROTACs, or nucleic acid therapeutics (83).

### 2.2 Metal nanocarriers

**Figure 1:**
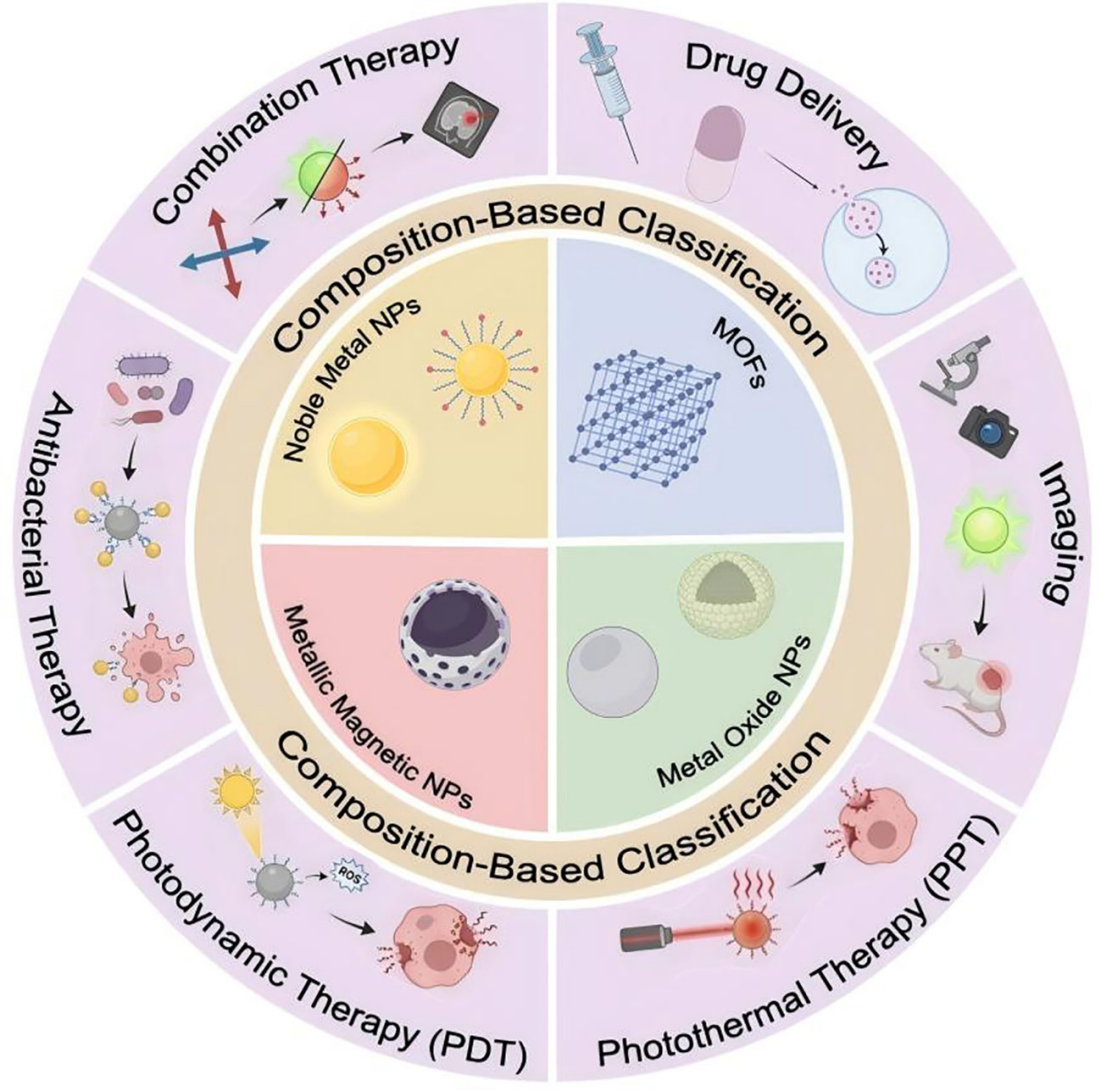
Different types of metal nanocarriers and their applications.

Metal-based nanocarriers exploit the optical, electrical, magnetic, and catalytic properties of metals or metal oxides for drug delivery, imaging, and combination therapy (**Figure 1**). According to composition, they can be broadly classified into noble-metal nanoparticles, transition-metal or metal-oxide nanoparticles, magnetic nanoparticles, and metal–organic frameworks (MOFs). Among noble-metal nanoparticles, gold nanoparticles are the most widely studied because their surface plasmon resonance enables near-infrared photothermal conversion, while their surface chemistry allows conjugation with drugs or targeting ligands. In TNBC, CD44-targeted gold–doxorubicin nanocomposites have been used for pulsed chemo-photothermal therapy, and cetuximab-functionalized gold nanorods have been applied to enhance EGFR-mediated uptake and photothermal ablation in TNBC spheroid models (84–86).

Silver nanoparticles also show anticancer and imaging potential, but their application requires careful control of Ag^+^ release and nonspecific toxicity. In TNBC models such as MDA-MB-231 cells, AgNPs have been reported to exert selective cytotoxicity and interact with doxorubicin, thereby influencing chemotherapeutic responses (87, 88).

Magnetic metal-oxide nanoparticles, especially Fe_3_O_4_-based platforms, are attractive because they can combine magnetic targeting, magnetic hyperthermia, imaging, and drug or nucleic acid delivery. In TNBC-related models, Fe_3_O_4_ and γ-Fe_2_O_3_ systems have been investigated for chemo-immunotherapy, gene delivery, and magnetic hyperthermia. However, these platforms should still be described as preclinical “magnetic navigation plus therapeutic delivery” systems rather than established TNBC therapies (89–92). Representative examples include doxorubicin-loaded magnetite nanoparticles, anti-EGFR-targeted magnetic nanocarriers for selective doxorubicin delivery, and vortex magnetic nanorods for the co-delivery of doxorubicin and EZH2-targeting siRNA (93, 94). The latter strategy integrates cytotoxic chemotherapy with epigenetic modulation, since EZH2 silencing may attenuate malignant programs associated with TNBC aggressiveness and sensitize tumor cells to doxorubicin (95). Thermosensitive magnetic hydrogels have also been used to asynchronously release doxorubicin and docetaxel to suppress TNBC growth and recurrence (96).

Overall, metal-based nanocarriers provide versatile platforms for imaging-guided therapy and combination treatment, but their translation remains limited by long-term retention, metal-ion release, oxidative stress, and quality-control requirements. In addition to iron oxide platforms, ZnO-based, CuO-based, and metal sulfide-based nanocarriers have been explored for drug delivery, photosensitizer delivery, photothermal therapy, or photodynamic therapy in TNBC (97, 98). MOFs represent another important class of metal-hybrid nanocarriers because their porous crystalline frameworks provide high surface area, tunable pore structures, and opportunities for ligand modification (99). For example, transferrin-modified UiO-66 co-loaded with doxorubicin and indocyanine green integrates chemotherapy, photothermal therapy, and photodynamic therapy within a single TNBC-oriented nanosystem (100). Other representative metal-oxide examples include PEGylated ZnO nanoparticles synthesized by an Aloe vera-mediated green method, which showed higher doxorubicin loading than gemcitabine loading and stronger cytotoxicity against MDA-MB-231 cells than unloaded or non-PEGylated controls (101). Folic acid-decorated CuO nanowires have also been reported to enhance tumor-cell uptake, induce mitochondrial ROS generation, modulate the NF-κB/miR-425/PTEN axis, promote apoptosis, and reduce the migratory potential of TNBC cells (102).

Metal nanocarriers can be prepared through wet chemical reduction, seed-mediated growth, coprecipitation, thermal decomposition, hydrothermal synthesis, sol-gel methods, solution combustion, green biosynthesis, or coordination-driven self-assembly, depending on their composition and intended function (103–106). For noble-metal and metal-oxide nanoparticles, parameters such as precursor concentration, reducing or stabilizing agents, pH, temperature, and reaction time regulate particle size, morphology, dispersity, colloidal stability, and optical, magnetic, or photothermal properties. Green biosynthetic strategies using plant extracts, polysaccharides, proteins, or microbial biomass have also attracted attention because these components may act as mild reducing and stabilizing agents and form surface-capping layers that improve colloidal stability and potentially reduce nonspecific toxicity (107). MOF-based carriers are generally prepared by solvothermal methods or room-temperature self-assembly, in which metal salts and organic ligands form nanometer-scale crystals through coordination-driven assembly. After formation, these carriers can accommodate cargos within their porous frameworks through pore confinement and non-covalent interactions such as electrostatic interactions, hydrogen bonding, hydrophobic interactions, or π-π stacking, and they can also load functional cargos, including anticancer agents and gaseous signaling molecules, through coordination interactions at the metal nodes (108).

Metal-based nanocarriers typically employ three strategies for drug loading (**Table 2**). First, drugs that are negatively charged or contain N/S/O coordinating atoms can be directly bound to metal or metal-oxide surfaces via electrostatic adsorption and coordination bonding. Second, an organic shell—such as silica, polymer, or lipid layers—can be constructed, and the drug is then encapsulated within the shell or at the core–shell interface. Third, covalent conjugation can be achieved by using surface carboxyl, amino, or thiol groups, or via click chemistry, to attach small-molecule drugs, nucleic acids, or peptides to the outer layer of the carrier, enabling controlled release and active targeting. In addition, metal nanocarriers are often integrated with stimulus-responsive mechanisms—such as pH, light, magnetic fields, or hydrogen peroxide—to trigger drug release or structural disassembly, thereby enabling “on-demand drug delivery” (115).

**Table 2:** Overview of preparation methods and drug encapsulation characteristics of representative common metal nanoparticles.

Carrier type	Preparation method	Loading capacity/characteristics	References
Gold nanoparticles (AuNPs)	Sodium citrate reduction; ligand exchange; microfluidic reduction; hard-template methods/electroplating to fabricate nanoshells/nanocages	Good biocompatibility; Au–S/Au–N coordination facilitates covalent conjugation of drugs/ligands; photothermal therapy and photoacoustic imaging	(109, 110)
Silver nanoparticles (AgNPs)	Chemical reduction; green (bio) synthesis; microemulsion/microfluidics	Strong antibacterial activity, but cytotoxicity and Ag^+^ release require control; often loaded via surface adsorption/coordination	(111, 112)
Superparamagnetic iron oxide nanoparticles (SPION)	Coprecipitation; microemulsion; hydrothermal synthesis	MRI tracking and magnetic targeting; after silanization or coating with polyacids/polysaccharides, drugs can be loaded via adsorption/covalent linkage	(113, 114)

### 2.3 Inorganic non-metallic nanocarriers

Inorganic non-metallic nanocarriers mainly include silica-based materials, calcium phosphate–based systems, and various carbon-based nanomaterials, among which mesoporous silica nanoparticles (MSNs) and carbon nanostructures (e.g., graphene, carbon nanotubes, and carbon dots) are the most representative. MSNs feature highly tunable pore sizes (typically 2–10 nm), ordered pore-channel architectures and large specific surface areas, enabling high drug-loading capacity and diverse stimulus-responsive gating. They are therefore widely used for delivering small-molecule drugs, proteins, and nucleic acids (116). Common MSN architectures include MCM-41, SBA-15, and a variety of morphologies such as spherical, rod-like, and worm-like forms. MCM-41 and SBA-15 are two typical types of ordered mesoporous silica materials: the former usually possesses smaller and more uniform pore sizes, making it suitable for the loading and controlled release of small-molecule drugs, whereas the latter has larger pores and thicker pore walls, which are more conducive to the loading of macromolecular drugs and the maintenance of structural stability (117). By functionalizing the inner and outer pore surfaces with amino, thiol, carboxyl groups, or polymer brushes, controlled release can be achieved in response to multiple cues, including pH, enzymes, and reductive microenvironments.

Stimuli-responsive gating introduces “switch”-like structures onto the surface or pore openings of inorganic nonmetallic nanocarriers that can respond to endogenous or exogenous tumor-associated stimuli, thereby keeping the drug relatively sealed during blood circulation while enabling selective release under conditions such as the acidic tumor microenvironment, the highly reductive intracellular milieu, or near-infrared irradiation. In this way, premature drug leakage and nonspecific exposure can be reduced, while the effective local drug concentration at the tumor site can be increased (118). Given the heterogeneity and limited therapeutic window of TNBC, as discussed above, this strategy of “stable transport first, followed by site-specific release” is particularly valuable (119).

Among various inorganic non-metallic nanoplatforms, mesoporous silica nanoparticles (MSNs) are one of the most mature systems for stimuli-responsive gating research because of their regular pore architecture, large specific surface area, and ease of surface functionalization (120). Their key advantage lies not only in their high drug-loading capacity, but also in the ability to seal pore openings with polymers, dynamic covalent bonds, or cleavable linkers, thereby enabling drug release to be regulated by tumor microenvironment-specific stimuli. In TNBC, for example, hyaluronic acid (HA)-modified mesoporous silica/hydroxyapatite hybrid nanoparticles (CL@M/H-HA) have been used for the co-delivery of cabazitaxel and the PI3K inhibitor LY294002. In this system, the hydroxyapatite component confers pronounced pH-responsive release behavior, while also enabling the synchronous and proportional release of the two agents, resulting in stronger pro-apoptotic and synergistic inhibitory effects in MDA-MB-231 cells. This indicates that the significance of stimuli-responsive gating in TNBC is not limited to reducing premature drug leakage; more importantly, it can provide a “synergistic release window” for combination therapy, thereby improving the spatiotemporal coordination of different therapeutic modules within the same lesion (121). In addition to mesoporous silica, calcium phosphate-based inorganic nonmetallic materials also exhibit considerable gating potential (122). These materials possess favorable biocompatibility and acid-responsive degradability, allowing them to function not only as carriers but also as self-degradable switches within the acidic tumor microenvironment (123). Their relevance is particularly evident in TNBC bone metastasis, because breast cancer commonly metastasizes to bone, liver, lung, and brain, and skeletal involvement can lead to bone pain, bone destruction, pathological fractures, and impaired quality of life (124). Calcium phosphate and hydroxyapatite resemble the inorganic mineral phase of bone, may favor retention in bone-related lesions, and can participate in the modulation of bone-resorption-associated microenvironmental changes (125). For instance, pH/redox dual-responsive calcium phosphate hybrid micelles (DZ@CPH), developed for TNBC bone metastasis, achieve docetaxel delivery to bone metastatic lesions through hybrid micelles reinforced with a calcium phosphate structure. This system can promote stimulus-responsive drug release, inhibit bone resorption-associated microenvironmental remodeling, reduce osteoclast activity, and improve local immunosuppression. These findings suggest that calcium phosphate-based gating strategies in TNBC are not limited to controlled release at the primary tumor site, but may also be applied to metastatic niches where tumor-cell killing and microenvironmental modulation are both required (126).

The preparation of MSNs primarily relies on sol-gel chemistry combined with soft- or hard-template strategies (127). In a typical modified Stöber process, alkoxysilanes such as tetraethyl orthosilicate undergo hydrolysis and condensation around surfactant or block-copolymer templates, followed by template removal to generate ordered mesoporous structures. By adjusting the template type, reaction conditions, and surface functionalization, MSN particle size, pore size, morphology, colloidal stability, and drug-release behavior can be tuned (128). Other approaches, including evaporation-induced self-assembly, microemulsion, hydrothermal or microwave-assisted synthesis, and hard-template replication, are also used to construct MSNs with different architectures for drug delivery and stimulus-responsive gating (129).

Calcium phosphate-based nanocarriers are commonly prepared by coprecipitation, hydrothermal or solvothermal processing, spray drying, or template-assisted mineralization. During preparation, pH, calcium/phosphate ratio, stabilizers, and surface modifiers such as citrate, poly(acrylic acid), proteins, or polymers can regulate particle morphology, surface charge, colloidal stability, degradability, and drug-loading behavior (130, 131). When calcium phosphate or hydroxyapatite is introduced as a functional component, loading efficiency should ideally be reported before and after mineral incorporation or surface modification whenever comparative data are available. For example, citrate-functionalized hydroxyapatite nanoparticles achieved approximately 85% doxorubicin loading efficiency at a drug-to-particle ratio of 1:10, which was attributed to electrostatic interactions between positively charged doxorubicin and negatively charged citrate-functionalized hydroxyapatite, together with the porous structure of the carrier (132). In another poly(acrylic acid)/polyethylene glycol/hydroxyapatite nanocomposite system, the reported doxorubicin loading and entrapment efficiencies reached 46% and 87.5%, respectively. These examples indicate that improved loading is not an intrinsic property of all calcium phosphate or hydroxyapatite systems, but depends on particle composition, surface functionalization, porosity, and the drug-to-carrier ratio (133).

Carbon-based nanocarriers constitute a major branch of inorganic non-metal systems and include zero-dimensional fullerenes and carbon dots, one-dimensional carbon nanotubes (CNTs), and two-dimensional graphene and its derivatives such as graphene oxide (GO) and reduced graphene oxide (rGO). Owing to its abundant carboxyl, hydroxyl, and epoxy groups, GO is readily amenable to covalent and noncovalent functionalization, can efficiently load aromatic small molecules and photosensitizers via π–π stacking and hydrophobic interactions, and can bind oligonucleotides and proteins through electrostatic or covalent approaches; consequently, it has been widely applied in anticancer and gene-delivery applications (134). Studies have shown that functionalized GO can be employed for chemo/gene co-delivery in TNBC (135) and for chemo-photothermal synergistic therapy (136). CNTs, by contrast, offer a tubular hollow architecture and excellent mechanical and electrical properties, making them well suited as long-circulating, cell-membrane-penetrating drug carriers and as scaffold materials (137). CD44-targeted multi-walled carbon nanotubes can enhance drug uptake and antitumor efficacy in MDA-MB-231 cells (138). Carbon dots provide another carbon-based platform with fluorescence, biocompatibility, surface modifiability, and drug-loading capacity. Anti-PD-L1-labeled carbon dots have demonstrated potential for TNBC imaging and immune-related therapy (139), whereas HA-modified carbon quantum dots have been reported to induce ferroptosis in TNBC (140). Overall, carbon-based nanocarriers provide multifunctional platforms for TNBC that combine drug delivery, imaging, and combination therapy, although their clinical translation still requires further resolution of biosafety and *in vivo* metabolism issues (141).

Carbon-based nanomaterials are generally synthesized through top-down or bottom-up strategies, including chemical vapor deposition(CVD), oxidative exfoliation, hydrothermal or microwave-assisted carbonization, and electrochemical methods, which influence their morphology, surface chemistry, fluorescence, drug-loading behavior, and biocompatibility (142). Owing to their fluorescence, surface modifiability, and drug-loading capacity, carbon dots have been explored as imaging-guided theranostic platforms in TNBC. For example, folic acid-based carbon dot-functionalized amphiphilic nanomicelles have been developed for targeted doxorubicin delivery and concurrent bioimaging in MDA-MB-231 TNBC cells, while gadolinium-doped carbon dots have been evaluated in 4T1 and MDA-MB-231 TNBC cells *in vitro* and in subcutaneous 4T1 tumor-bearing mouse models for MRI-guided drug delivery and NIR-triggered photothermal chemotherapy (143–145). BP nanosheets are usually obtained by exfoliation and require surface stabilization because they are prone to oxidative degradation in aqueous and oxygenated environments (146).

Overall, the preparation of inorganic non-metallic nanocarriers mainly involves top-down exfoliation or etching, as well as bottom-up approaches such as sol–gel synthesis, vapor deposition, and hydrothermal methods (**Table 3**). Through fine control over size, morphology, and surface chemistry, multifunctional platforms can be obtained. These platforms combine high loading capacity, good biocompatibility, and designable stimulus-responsive behaviors, providing a rich material basis for improving the dissolution of poorly soluble drugs, enabling targeted delivery, and developing integrated theranostic systems (159).

**Table 3:** Overview of preparation methods and drug encapsulation characteristics of inorganic non-metallic nanoparticles.

Carrier type	Preparation method	Loading capacity/characteristics	References
Mesoporous silica	Soft templating; hard-template replication; template removal	Pore adsorption; gatekeeper (“valve”) control; suitable for small molecules/peptides	(147, 148)
Hydroxyapatite/calcium phosphate	Coprecipitation; solvothermal/hydrothermal synthesis; template-induced mineralization	Surface adsorption/ion exchange; suitable for proteins, peptides, and genes	(149, 150)
Carbon dots	Hydrothermal/microwave synthesis; pyrolysis/carbonization; laser ablation; electrochemical exfoliation	Covalent/noncovalent loading; imaging-enabled delivery	(151, 152)
Carbon nanotubes (CNTs)	Chemical Vapor Deposition; arc discharge; laser ablation; oxidative cutting/shortening; PEGylation	Lumen/surface adsorption with π–π interactions; suitable for hydrophobic drugs/siRNA	(153, 154)
Black phosphorus (BP)	Liquid-phase ultrasonication/shear exfoliation; electrochemical exfoliation; surface coating	Physical adsorption/covalent conjugation; photothermal/photosensitization synergy	(155, 156)
Graphene/graphene oxide (GO)	Hummers method; liquid-phase exfoliation; Chemical Vapor Deposition	Sheet-surface adsorption/covalent grafting; combined photothermal/photosensitization applications	(157, 158)

### 2.4 Other types of nanocarriers

In addition to protein-, metal-, and inorganic non-metallic nanocarriers, polymeric nanoparticles, nanogels, dendrimers, lipid-based nanocarriers, bio-derived nanocarriers, and hybrid intelligent nanocarriers also constitute important components of TNBC nanotherapeutic research and remain among the most active areas in both preclinical investigation and clinical translation (160). Because TNBC exhibits substantial molecular heterogeneity, lacks well-defined therapeutic targets, and is prone to recurrence and metastasis, effective treatment often requires combination strategies. These features make nanocarrier systems particularly attractive for improving drug delivery, enabling nucleic acid transport, modulating antitumor immunity, and supporting theranostic applications (161).

Among the various platforms, polymeric nanocarriers constitute a major class, including biodegradable polyester nanoparticles, polymeric micelles, nanogels, and dendrimers. Nanoparticles, such as those of PLA, PLGA, and PCL, exhibit good biocompatibility and controllable degradability, and are commonly used in TNBC for loading taxanes, doxorubicin, and hydrophobic molecularly targeted agents, thereby improving drug stability, prolonging circulation time, and reducing systemic toxicity (162). These nanoparticles are usually prepared by the emulsification-solvent evaporation method or nanoprecipitation. In the former, the polymer and drug are dissolved in an organic phase, dispersed into an aqueous phase to form an emulsion, and then the organic solvent is removed to obtain drug-loaded nanoparticles. In the latter, the polymer and drug are first co-dissolved in a water-miscible organic solvent such as acetone, acetonitrile, ethanol, or dimethyl sulfoxide (163). When this organic phase is added to an aqueous phase, rapid solvent exchange decreases polymer and drug solubility, driving polymer self-assembly into nanoparticles while hydrophobic drugs partition into the hydrophobic core or become trapped within the forming polymer matrix (163). The residual organic solvent is then removed, and the drug-loaded nanoparticles are purified to eliminate free drug and solvent residues (164). This process is relatively simple and easy to scale up (165). Polymeric micelles are formed by the self-assembly of amphiphilic block copolymers in aqueous solution, yielding a “hydrophobic core-hydrophilic shell” structure. They are particularly suitable for the delivery of hydrophobic chemotherapeutic agents and small-molecule inhibitors in TNBC. Further incorporation of targeting ligands or pH-/redox-responsive segments can also enable active targeting and stimuli-responsive release in TNBC (166). Nanogels, by contrast, are three-dimensional networks formed by chemical or physical crosslinking of hydrophilic polymers and are suitable for loading proteins, nucleic acids, and hydrophilic small molecules. In TNBC, they are commonly used for the delivery of siRNA, miRNA, or immunomodulatory agents, and are generally prepared by inverse emulsion polymerization, photocrosslinking, or click chemistry (167). Dendrimers, owing to their highly branched architecture and abundant surface functional groups, can achieve co-delivery of small-molecule drugs and nucleic acids in TNBC and are advantageous for multivalent modification and functional integration (168).

Lipid-based nanocarriers are commonly fabricated by thin-film hydration, ethanol injection, reverse-phase evaporation, or high-pressure homogenization, depending on whether liposomes, solid lipid nanoparticles (SLNs), nanostructured lipid carriers (NLCs), or ionizable lipid nanoparticles (LNPs) are being prepared. Bio-derived carriers such as EVs and cell membrane-coated nanoparticles require additional control over source cells, isolation methods, cargo loading, membrane coating, and batch consistency, which are particularly important for future clinical translation (169, 170).

In addition to these artificially synthesized systems, bio-derived nanocarriers have also demonstrated unique advantages in TNBC, mainly including exosomes/extracellular vesicles, bacteria-derived nanovesicles, and cell membrane-coated nanoparticles (171). Because exosomes originate from natural cellular secretion, they generally exhibit low immunogenicity and good biocompatibility. Their endogenous targeting capability mainly refers to parental-cell-dependent tropism and preferential uptake by certain recipient cells, which are mediated by surface molecules such as integrins, tetraspanins, adhesion proteins, and glycans. In TNBC management, this property is relevant because vesicles derived from tumor cells, immune cells, or stromal cells may preferentially interact with homologous tumor cells, immune-cell subsets, or inflammation-associated metastatic niches, thereby improving delivery to primary tumors or disseminated lesions while reducing nonspecific exposure. However, this targeting is relative and context-dependent rather than absolutely specific (172, 173). In TNBC, exosomes are commonly used for the delivery of siRNA, miRNA, chemotherapeutic agents, or immunomodulatory molecules, and are especially suitable for crossing biological barriers and delivering drugs to metastatic lesions (174). They are usually isolated from cell culture supernatants or body fluids by ultracentrifugation, density gradient centrifugation, or size-exclusion chromatography, and drugs can then be loaded by incubation, electroporation, or sonication (175). Cell membrane-coated nanoparticles are prepared by extracting membranes from red blood cells, platelets, or tumor cells and then coating them onto polymeric or inorganic nanoparticle cores, thereby endowing the carrier with “self” camouflage, immune evasion, and homologous targeting properties (176). In TNBC, tumor cell membrane-coated systems are particularly suitable for enhancing the recognition of primary tumors and metastatic lesions, whereas platelet membrane- or macrophage membrane-coated systems are more appropriate for targeting inflammation-associated microenvironments. These systems are typically fabricated by membrane isolation followed by extrusion or sonication-induced fusion (177).

Furthermore, hybrid and intelligent nanocarriers represent an important developmental direction in TNBC nanotherapy. By organically integrating different components such as polymers, lipids, inorganic materials, and biomembranes, these systems can be engineered into composite platforms with multiple stimuli-responsive features and multifunctional synergistic effects, including lipid-polymer hybrid nanoparticles, protein-inorganic hybrid nanocages, and membrane-coated intelligent nanoparticles (**Table 4**) (190). In TNBC, such carriers are often used to integrate chemotherapy, photothermal/photodynamic therapy, immunomodulation, and imaging functions within a single platform, while enabling on-demand drug release and spatiotemporal control through pH, enzyme, redox, or light responsiveness (191). Their preparation methods generally rely on stepwise self-assembly, core-shell assembly, interfacial deposition, or microfluidic-assisted hybrid fabrication. Overall, these “other types” of nanocarriers not only broaden the strategies available for drug delivery in TNBC, but also provide more flexible technological platforms for nucleic acid therapy, combination therapy, and individualized precision intervention (192).

**Table 4:** Overview of preparation methods and drug encapsulation characteristics of miscellaneous nanoparticles.

Carrier type	Preparation method	Loading capacity/characteristics	References
Biodegradable polyester nanoparticles (PLA/PLGA/PCL)	Emulsification–solvent evaporation; nanoprecipitation	Primarily load hydrophobic small molecules; high encapsulation efficiency; controlled release; simple and scalable manufacturing; surface PEGylation and targeting functionalization feasible	(178, 179)
Polymeric micelles (amphiphilic block copolymers)	Aqueous self-assembly; solvent displacement/exchange; thin-film hydration; core/shell crosslinking; ligand grafting or incorporation of pH-/redox-responsive segments	Hydrophobic drugs partition into the hydrophobic core; hydrophilic corona enables long circulation and hemocompatibility; amenable to active targeting and multistage stimulus responsiveness	(180, 181)
Nanogels	Inverse emulsion polymerization; photocrosslinking; chemical crosslinking; physical crosslinking (ionic/temperature/pH)	Suitable for proteins, nucleic acids, and hydrophilic small molecules; high water content enables high payloads; release can be triggered by temperature, pH, enzymes, etc.	(182, 183)
Liposomes	Thin-film hydration followed by sonication/extrusion; ethanol injection; reverse-phase evaporation	Hydrophilic drugs encapsulated in the aqueous core and hydrophobic drugs in the lipid bilayer; clinically mature with controllable size; PEGylation can prolong circulation	(184, 185)
Solid lipid nanoparticles (SLNs)	High-pressure homogenization; microemulsion method; melt emulsification + ultrasonication	Solid-lipid core improves drug stability; suitable for hydrophobic drugs; enables sustained release	(186, 187)
Exosomes/extracellular vesicles (EVs)	Isolation by ultracentrifugation, density-gradient centrifugation, or size-exclusion chromatography; loading via electroporation/incubation/sonication	Autologous-like composition with low immunogenicity and intrinsic targeting; can carry small molecules/proteins/nucleic acids; source selection and scalable production require further optimization	(188, 189)

Based on the above classification of nanocarrier systems, a comparative evaluation is necessary to clarify the relative strengths, limitations, TIME-modulating mechanisms, and translational status of different platforms in TNBC. Therefore, **Table 5** summarizes the major nanocarrier types, targeting mechanisms, therapeutic cargos, immune-regulatory effects, limitations, and clinical status.

**Table 5:** Comparative evaluation of major nanocarrier systems for TIME modulation in TNBC.

Nanocarrier type	Targeting mechanism	Representative cargos/strategies	TIME-modulating efficacy	Main limitations	Clinical status	References
Protein-based nanocarriers	Receptor-mediated uptake, especially albumin-, ferritin-, EGFR-, or TfR1-related pathways	Paclitaxel, doxorubicin, siRNA, PROTACs, immune modulators	Improve drug stability, tumor uptake, and intracellular delivery; may enhance antigen release or immune sensitization	Possible immunogenicity, limited loading flexibility, protein stability issues, source-dependent variability	Albumin-based formulations are clinically used; most immune-modulating systems remain preclinical	(193, 194)
Metal and metal-oxide nanocarriers	Surface ligand targeting, magnetic guidance, photothermal or catalytic responsiveness	DOX, siRNA, photosensitizers, photothermal agents, imaging probes	Enable imaging-guided therapy, photothermal/photodynamic immune activation, and combination treatment	Long-term retention, metal ion release, oxidative stress, unclear chronic toxicity	Mainly preclinical in TNBC; some iron oxide-based platforms have broader clinical experience	(195, 196)
Metal-organic frameworks	Porous confinement, ligand modification, pH/redox-responsive degradation	Chemotherapeutics, photosensitizers, gas molecules, immunomodulators	High loading capacity and multi-cargo delivery; can combine chemotherapy, phototherapy, and immune modulation	Structural stability, biodegradation, metal/ligand safety, complex quality control	Mostly preclinical	(197, 198)
Inorganic non-metallic nanocarriers	Pore gating, pH/redox responsiveness, bone-mineral affinity, photothermal conversion	DOX, docetaxel, cabazitaxel, PI3K inhibitors, photosensitizers	Support stimulus-responsive release and combination therapy; calcium phosphate/hydroxyapatite may be useful in bone metastatic niches	Biodegradation varies by material; possible long-term accumulation; limited clinical validation	Mostly preclinical	(199, 200)
Polymeric nanoparticles and micelles	Passive accumulation, ligand targeting, pH/redox/enzyme-responsive release	Taxanes, anthracyclines, small-molecule inhibitors, siRNA, immune modulators	Improve solubility, circulation time, controlled release, and combination delivery; may remodel CAFs, TAMs, or immune checkpoints	Premature drug leakage, batch variability, polymer degradation products, scale-up issues	Some polymeric nanomedicines have entered clinical use in oncology; TNBC TIME-targeted systems remain mostly preclinical	(201, 202)
Lipid-based nanocarriers	Membrane fusion, endocytosis, ligand targeting, ionizable lipid-mediated nucleic acid delivery	DOX, paclitaxel, siRNA, mRNA, CRISPR-related cargos, immunomodulators	Strong potential for nucleic acid delivery and immune reprogramming; compatible with ICB combinations	Stability, liver accumulation, infusion reactions, PEG-related immune responses	Clinically mature as a platform, but TNBC-specific TIME-targeted applications are still developing	(203, 204)
Extracellular vesicles and biomimetic nanoparticles	Homologous targeting, immune-cell tropism, membrane-mediated immune evasion	siRNA, miRNA, chemotherapeutics, checkpoint modulators, tumor antigens	May improve tumor or immune-cell targeting, metastatic niche recognition, and immune modulation	Heterogeneous composition, difficult standardization, low yield, uncertain biodistribution	Early-stage preclinical or exploratory clinical development	(205, 206)
Hybrid/intelligent nanocarriers	Multi-ligand targeting, sequential release, multi-stimulus responsiveness	Chemo-immunotherapy combinations, phototherapy agents, vaccines, checkpoint modulators	Integrate tumor killing, immune activation, imaging, and microenvironment remodeling in one platform	Excessive design complexity, difficult manufacturing, regulatory uncertainty, reproducibility concerns	Mostly preclinical; clinical translation requires simplification	(207, 208)

## 3 Research advances in nanocarriers targeting the tumor microenvironment in TNBC

### 3.1 Nanoimmunotherapy in TNBC

**Figure 2:**
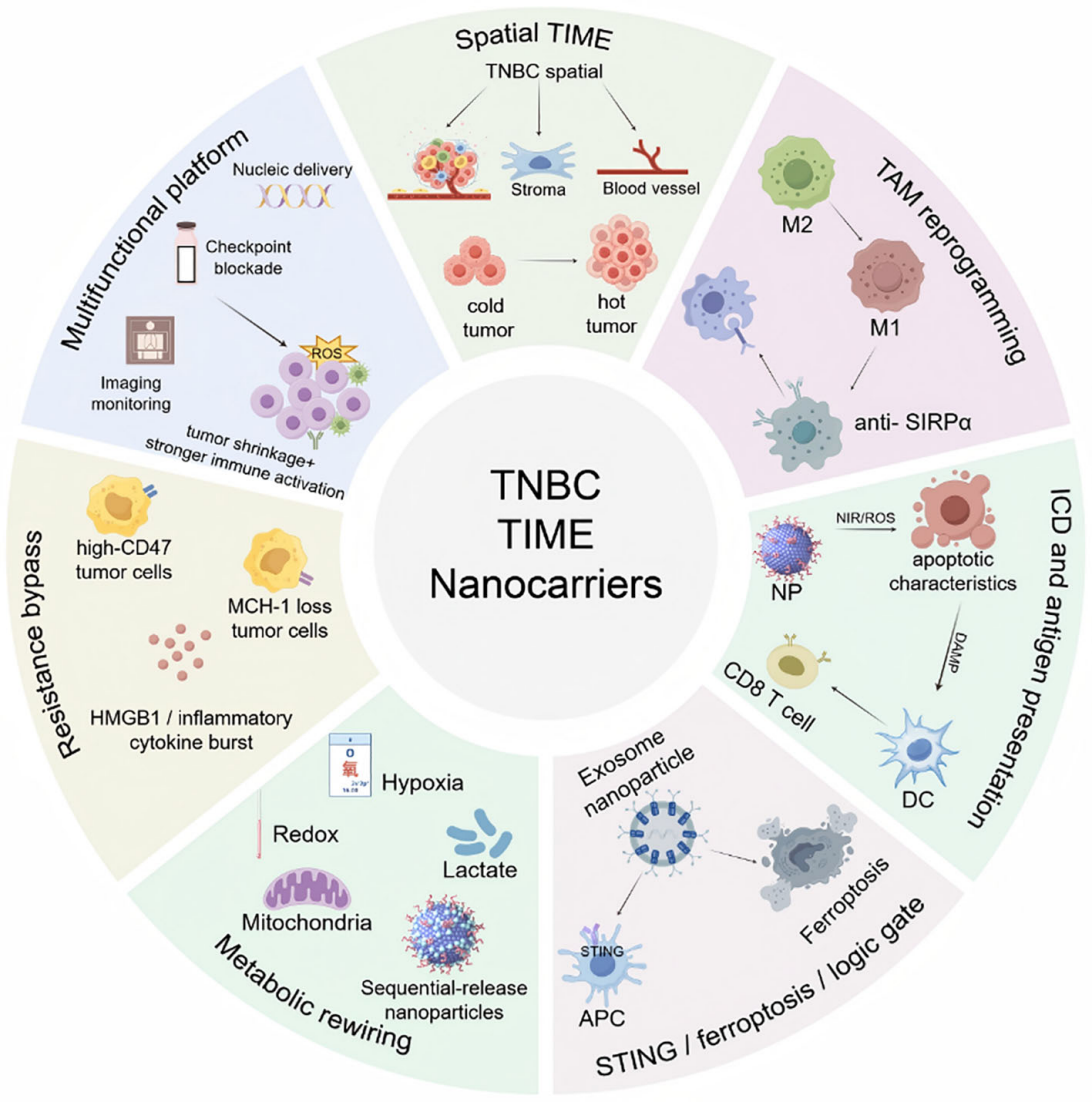
Research advances in nanocarriers targeting the tumor microenvironment in TNBC.

TNBC is characterized by high invasiveness, a strong tendency for recurrence and metastasis, and pronounced molecular heterogeneity (**Figure 2**). Compared with other breast cancer subtypes, TNBC generally exhibits higher levels of tumor-infiltrating lymphocytes (TILs) and more active expression of immune-related genes, and is therefore considered one of the breast cancer subtypes with the greatest potential to benefit from immunotherapy. However, in clinical practice, immune checkpoint blockade (ICB) benefits only a subset of patients with TNBC, largely because the TIME in TNBC is highly complex and markedly heterogeneous (209). This complexity involves not only CD8+ T cells, DCs, and NK cells that promote antitumor immunity, but also an abundance of TAMs, MDSCs, Tregs, and immunosuppressive cancer-associated fibroblasts (CAFs), thereby creating a state in which immune evasion, chronic inflammation, metabolic competition, and stromal barriers coexist (210). In TNBC, a dense stromal matrix, CAF activation, and elevated tissue tension are major barriers to effective nanodrug delivery and the sensitization of immunotherapy. By secreting collagen, fibronectin, and profibrotic factors, CAFs promote matrix stiffening, vascular compression, and restricted infiltration of effector T cells, making them an important target for TIME remodeling. To address this issue, one study loaded tranilast, an antifibrotic mechanotherapeutic agent, into PEG-PBLG [poly(ethylene glycol)-block-poly(γ-benzyl-L-glutamate)] polymeric micelles, thereby more effectively reprogramming CAFs, reducing tumor stiffness, relieving vascular compression, and improving nanomedicine penetration in TNBC. Composed of PEG and PBLG, these micelles exhibit favorable drug-loading capacity, circulation stability, and tumor accumulation. Compared with the free drug, the micellar formulation more effectively reprogrammed CAFs at approximately a 100-fold lower dose, reduced tumor stiffness, relieved vascular compression, improved perfusion, and enhanced the penetration and dispersion of nanomedicines within tumors (211).

In recent years, spatial omics and single-cell studies have further demonstrated that the TIME of TNBC is not merely a simple accumulation of cells, but rather a highly organized system with distinct spatial architecture and functional stratification. Using multiplexed ion beam imaging, Keren et al. revealed that tumor cells, stromal cells, and immune cells in TNBC are arranged in an ordered spatial structure, and that these spatial relationships are closely associated with patient prognosis (212, 213). Wang et al. further found that cellular phenotype, activation status, and spatial location jointly influence the response to immune checkpoint blockade, suggesting that the conversion of “cold tumors” into “hot tumors” depends not only on the number of immune cells, but also on their effective recruitment, activation, and interaction patterns within the tissue (214). Therefore, systemic administration alone is often insufficient to simultaneously overcome poor drug delivery efficiency, inadequate immune activation, and off-target toxicity. By contrast, nanocarriers, owing to their advantages in tumor accumulation, co-delivery, and microenvironment-responsive release, have gradually become important tools for remodeling the immune microenvironment of TNBC (215). Representative clinical-stage nanomedicine-related approaches relevant to TNBC or TME/TIME modulation are summarized in **Table 6**.

**Table 6:** Representative clinical-stage nanomedicine-related approaches relevant to TNBC or TME/TIME modulation.

Nanomedicine-related approach	Main formulation/target	Clinical relevance to TNBC or TME/TIME modulation	Current status	Representative trial/phase	References
Albumin-bound paclitaxel	Nab-paclitaxel; albumin-mediated taxane delivery	Evaluated in metastatic TNBC chemotherapy regimens and frequently used as a chemotherapy backbone in combination strategies; mainly improves drug formulation and delivery rather than directly remodeling a defined TIME component	Clinically used; phase II/III evidence in metastatic TNBC	Gradishar et al., phase III metastatic breast cancer trial; nab-paclitaxel plus cisplatin, randomized phase III mTNBC trial	(216, 217)
Pegylated liposomal doxorubicin	PEGylated liposomal anthracycline	Developed to reduce anthracycline-associated systemic and cardiac toxicity; evaluated in metastatic breast cancer and TNBC-related clinical settings	Clinically used in oncology; TNBC-specific studies remain limited	PLD in metastatic TNBC, retrospective clinical cohort; TNBC-specific prospective randomized trials remain limited	(218, 219)
Cationic liposomal paclitaxel	EndoTAG-1; tumor endothelial/angiogenic vasculature targeting	Represents a vascular/TME-oriented liposomal paclitaxel strategy; evaluated in advanced TNBC but not widely established as standard therapy	Clinical-stage/phase II evidence; limited later-stage validation	EndoTAG-1, randomized phase II trial in advanced TNBC	(220, 221)
Nanomedicine plus ICB	Nab-paclitaxel- or liposomal-drug-based regimens combined with PD-1/PD-L1 blockade	Links nanodrug formulation with immunotherapy; however, most clinical regimens use nanomedicine as chemotherapy backbone rather than as a specifically engineered TIME-remodeling platform	Clinically evaluated as chemotherapy backbone; mechanism-specific nano-ICB platforms remain preclinical	IMpassion130, phase III atezolizumab plus nab-paclitaxel; KN046 plus nab-paclitaxel, multicenter phase II mTNBC trial	(222, 223)
Mechanistically TIME-targeted nanocarriers	Nanocarriers targeting TAMs, MDSCs, Tregs, CAFs, STING, adenosine signaling, or immune exclusion	Strong preclinical rationale; no mature TNBC-specific clinical trial	Mostly preclinical or early exploratory development	No mature TNBC-specific clinical trial; mainly preclinical or early exploratory development	(224, 225)

**Figure 3:**
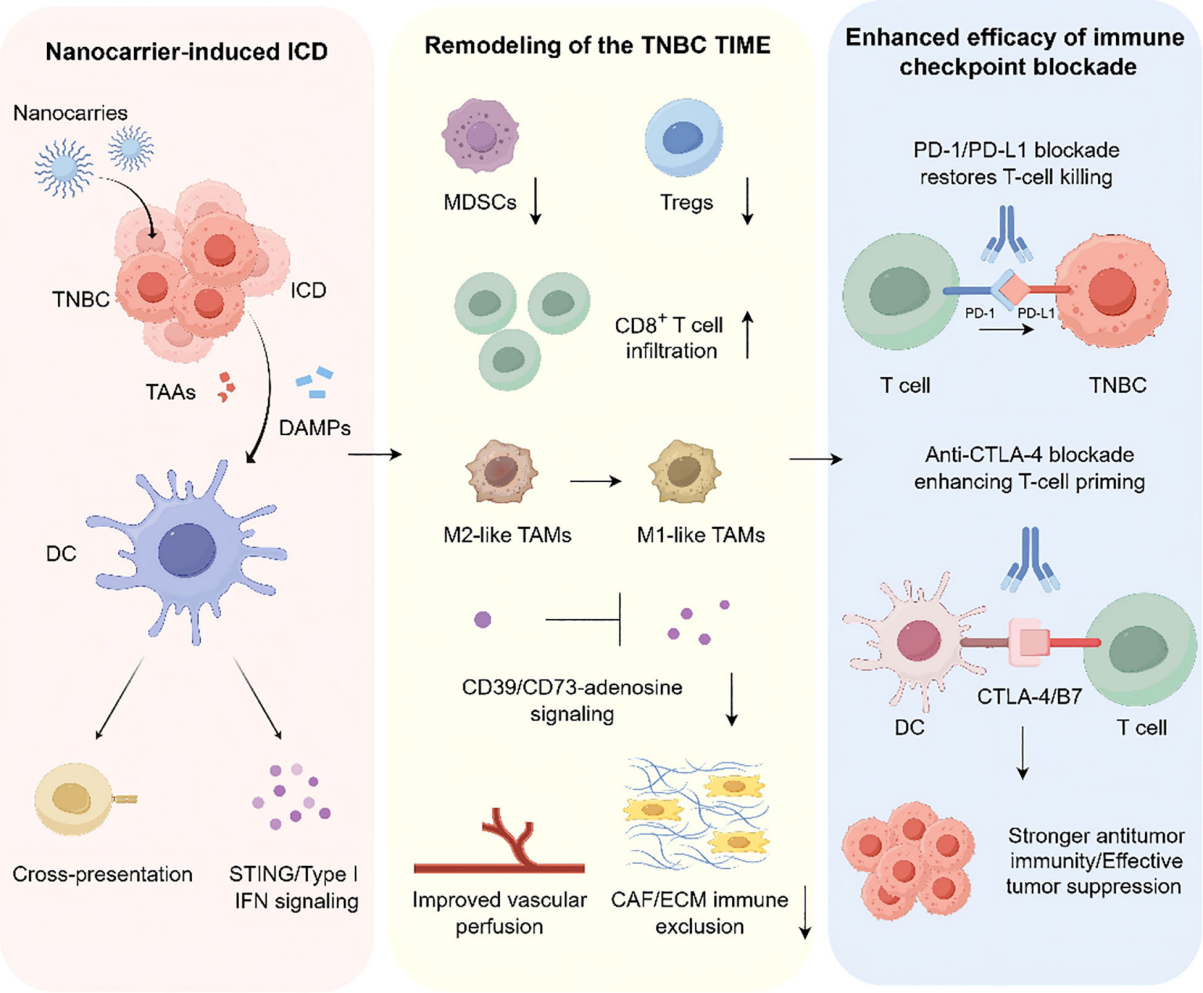
Proposed mechanistic illustration of nanocarrier-enhanced immune checkpoint blockade in TNBC.

The rationale for combining nanocarriers with ICB is not limited to increasing the local concentration of PD-1/PD-L1 or CTLA-4 inhibitors (**Figure 3**). More fundamentally, nanocarriers can alter the immune context required for checkpoint blockade to function (9). Chemotherapy-, phototherapy- or ferroptosis-inducing nanoplatforms may increase immunogenic cell death, release tumor-associated antigens and damage-associated molecular patterns, and promote dendritic-cell maturation and cross-presentation (226). These upstream events can increase the pool of tumor-reactive T cells that can subsequently be reinvigorated by checkpoint blockade (227).

Nanocarriers may also enhance ICB responsiveness by relieving suppressive barriers within the TNBC TIME. Examples include reprogramming M2-like TAMs toward inflammatory phenotypes, reducing MDSC and Treg-mediated suppression, activating the STING/type I interferon axis, disrupting CD39/CD73-mediated adenosine signaling, improving vascular perfusion, and decreasing CAF- or ECM-driven immune exclusion (228). These mechanisms suggest that nano-ICB strategies should be evaluated by immune remodeling endpoints such as DC activation, CD8+ T-cell infiltration, T-cell exhaustion status, myeloid polarization and spatial immune access, rather than by tumor volume alone (229).

However, the same immune-activating properties may also increase systemic inflammatory toxicity if spatial control is inadequate. Therefore, future nano-ICB strategies should balance immune activation with safety by optimizing release kinetics, tumor selectivity, dose schedule, and patient selection based on PD-L1/TIL status, immune-excluded architecture, myeloid abundance and stromal barriers (230).

### 3.2 Remodeling the tumor immune microenvironment in TNBC

#### 3.2.1 Nanostrategies targeting tumor-associated macrophage reprogramming and restoration of phagocytic function

Research on nanocarriers targeting the immune microenvironment of TNBC has initially focused on TAM reprogramming and restoration of phagocytic function. TAMs are among the most critical immunosuppressive cell populations in TNBC and are typically skewed toward an M2-like phenotype, thereby promoting angiogenesis, stromal remodeling, and T-cell exhaustion (231). To address this issue, researchers have developed a variety of nanosystems capable of simultaneously enhancing macrophage phagocytosis and inducing polarization switching from the M2 to the M1 phenotype. For example, the engineered nanoparticles constructed by Zhao et al. co-deliver an anti-CD24 antibody, celastrol, and MFN1-shRNA, and by blocking the CD24–Siglec10 “don’t eat me” signal while regulating mitochondrial dynamics in TAMs, they significantly enhance phagocytosis, antitumor immune responses, and postoperative immune memory in TNBC (232). Similarly, nanosystems based on the co-delivery of R848 and anti-SIRPα antibodies have also demonstrated the ability to synergistically promote macrophage phagocytosis and repolarization, providing a promising strategy for TNBC nano-immunotherapy centered on innate immunity (233).

#### 3.2.2 Remodeling of the immune microenvironment based on the STING pathway, ferroptosis, and metabolic regulation

In recent years, nanoplatforms centered on the STING pathway, ferroptosis, and metabolic intervention have also developed rapidly. The “logic-gated” Trojan-horse system proposed by Guo et al. employs an exosome-like structure carrying DNA fragments together with biodegradable hollow mesoporous organosilica nanoparticles, enabling differential activation of TNBC cells and antigen-presenting cells, thereby selectively inducing ferroptosis and activating the STING pathway (234). Meanwhile, Ye et al. coupled photothermal therapy, ferroptosis, and metformin-mediated immunoregulation through an Fe-PDA-MET nanoplatform, significantly increasing the infiltration of CD8+ T cells and NK cells and promoting the conversion of the TIME from an immune-cold to an immune-hot state (235).

#### 3.2.3 Nano-interventions targeting immunosuppressive metabolic networks

A third strategy emphasizes precise interventions in immunosuppressive metabolic networks and refractory cell subpopulations. Enhanced glycolysis, lactate accumulation, hypoxia, and redox imbalance are commonly observed in TNBC. These factors not only impair the functions of effector T cells and NK cells, but also promote the expansion of TAMs, MDSCs, and Tregs (236). The adenosine axis represents another important immunometabolic pathway contributing to the suppressive TIME of TNBC. Extracellular ATP released from stressed or dying tumor cells can be sequentially converted into AMP and adenosine by CD39 and CD73. Accumulated adenosine then activates A2A and A2B receptors on immune cells, thereby suppressing CD8^+^ T-cell and NK-cell effector functions, promoting regulatory or suppressive immune phenotypes, and weakening antitumor immunity (237). In TNBC, CD73 expression has been associated with immune escape, poorer clinical outcomes, and resistance to anthracycline-based therapy, suggesting that CD39/CD73-mediated adenosine signaling may represent a relevant target for TIME remodeling (238). Site-specific sequential release nanoparticles developed by She et al. achieve dual metabolic inhibition through stepwise interference with glycolysis and mitochondrial energy metabolism (239). After tumor-cell internalization, the reductive intracellular environment triggers the release of a CRISPR/Cas9 module that downregulates LDHA expression, thereby suppressing glycolysis-associated lactate production (240). Subsequently, CPI-Z2 is released from the nanoplatform to block mitochondrial tricarboxylic acid cycle activity (241). By simultaneously limiting lactate-dependent immunosuppression and mitochondrial energy metabolism, this strategy may remodel the immunosuppressive TNBC microenvironment and promotes antitumor immune activation (242).

#### 3.2.4 Precision nanotherapies for immune evasion and treatment-refractory TNBC subtypes

For TNBCs lacking MHC-I expression or showing poor responsiveness to anti-PD-L1 therapy, the LCL161-loaded macrophage membrane-coated nanoparticles constructed by Zhang et al. can recognize tumor cells with high CD47 expression via SIRPα. Their therapeutic value lies in localized immune activation rather than nonspecific systemic inflammation. On the one hand, LCL161-induced release of pro-inflammatory cytokines and HMGB1 can provide danger-associated signals that promote immunogenic tumor-cell stress, phagocyte activation, and antigen presentation. On the other hand, macrophage membrane decoration supports tumor recognition and enhances phagocytic activation, thereby partially overcoming the limitations of conventional CTL-dependent immunotherapy in MHC-I-deficient TNBC (243).

Overall, nanocarriers targeting the tumor immune microenvironment of TNBC have evolved from early single-function drug delivery tools designed merely to enhance drug accumulation into multifunctional platforms integrating precise targeting, immune remodeling, imaging monitoring, and combination therapy. Their core value is no longer limited to the delivery of chemotherapeutic agents, but rather lies in their ability to systematically improve the immunosuppressive niche of TNBC by regulating TAMs, DCs, T cells, CD39/CD73-mediated adenosine signaling, the STING pathway, and tumor metabolic networks, thereby enhancing the synergistic efficacy of immune checkpoint inhibitors, phototherapy, chemotherapy, and nucleic acid-based therapies.

### 3.3 Contradictory findings and unsuccessful translational outcomes

Although many TNBC nanoplatforms show strong antitumor activity in cell-derived xenograft, syngeneic or orthotopic mouse models, these positive results should be interpreted cautiously (244). Conflicting outcomes may arise from differences in tumor implantation site, immune competence, nanoparticle dose, administration route, particle size, surface charge, protein corona formation, stromal density and the selected efficacy endpoints (245). In particular, subcutaneous models may overestimate drug penetration and EPR-mediated accumulation compared with heterogeneous human TNBC lesions (246).

Several factors may explain why successful preclinical nanomedicines fail to progress clinically. These include over-reliance on passive EPR accumulation, insufficient validation in immunocompetent and metastatic models, lack of standardized immune endpoints, inadequate long-term toxicity assessment, excessive structural complexity, poor manufacturability, and the absence of biomarker-based patient selection (247). Therefore, negative or inconsistent findings should not be viewed merely as technical failures but as important evidence that TNBC nanomedicine requires model selection, mechanistic validation and clinical stratification (248).

### 3.4 Clinical-trial landscape of nanomedicine-related strategies in TNBC

Clinical translation of TME/TIME-targeted nanocarriers in TNBC remains at an early stage (249). Some clinically used or clinical-stage nanomedicine-related approaches, such as albumin-bound paclitaxel or pegylated liposomal doxorubicin, have been evaluated in breast cancer or TNBC treatment regimens (250). However, most platforms specifically designed to remodel TAMs, MDSCs, Tregs, stromal barriers, adenosine signaling or STING pathways remain preclinical. The distinction between clinically used nanodrug formulations and mechanistically TME-targeted nanocarriers is therefore important (251).

## 4 Challenges and future perspectives

Despite rapid progress in TNBC nano-immunotherapy, several barriers continue to limit its clinical translation. These include the structural complexity of multifunctional nanoplatforms, insufficient batch-to-batch reproducibility, difficulties in accurately predicting their biodistribution and long-term *in vivo* fate, and the substantial gap between simplified animal models and the immunological heterogeneity observed in patients with TNBC (252). Protein corona formation may alter nanoparticle identity, biodistribution, cellular uptake, immune recognition, and targeting efficiency, thereby complicating the extrapolation from *in vitro* performance to *in vivo* efficacy (253). Although patient stratification has been emphasized in this review as a key translational principle, it has not yet been fully standardized for TNBC nanomedicine (254). Future studies should therefore translate this principle into operational criteria, including immune subtype, PD-L1 and TIL status, stromal exclusion, receptor expression such as EGFR, TfR1, or folate receptor, EPR-related vascular features, and protein-corona behavior (255, 256). On this basis, clinically translatable materials, simplified and reproducible carrier designs, spatial multi-omics-guided precision engineering, and validation in combination with standard TNBC therapies should be prioritized. These efforts may help move TNBC nano-immunotherapy from proof-of-concept studies toward clinically applicable therapeutic strategies (252).

### 4.1 Clinical limitations and controversies of the EPR effect

The EPR effect remains an important conceptual basis for nanomedicine, but it should not be treated as a universally reliable delivery mechanism in patients (257). Human TNBC lesions differ markedly in vascular density, perfusion, endothelial permeability, interstitial pressure, stromal composition and prior treatment exposure. As a result, EPR-dependent accumulation may vary not only between patients but also between primary tumors, lymph-node metastases and visceral or bone metastases within the same patient (258). This limitation helps explain why passive targeting often performs better in murine models than in clinical settings.

Therefore, future TNBC nanotherapy should move beyond EPR-centric design. More clinically realistic strategies include combining passive accumulation with active receptor targeting, stromal or vascular modulation, image-guided assessment of tumor deposition, stimulus-responsive release, and patient selection based on vascular and stromal features (259).

### 4.2 Long-term toxicity, biodistribution and safety concerns

Long-term safety remains a central barrier to nanomedicine translation. Nanoparticles may accumulate in the liver, spleen, bone marrow and mononuclear phagocyte system, while renal clearance is strongly influenced by size, charge and degradation behavior (260). Persistent inorganic components, metal ion release, complement activation, hemolysis, chronic inflammation, immunogenicity and unexpected interactions with immune cells should be systematically evaluated (261). Thus, the term biocompatible should be used cautiously and only in relation to a defined material, dose, route, degradation profile and observation period (262).

### 4.3 Regulatory, manufacturing, quality-control and commercialization barriers

Regulatory translation requires more than proof of antitumor efficacy. Critical quality attributes such as particle size, polydispersity index, zeta potential, morphology, drug-loading efficiency, encapsulation efficiency, release profile, residual solvent, endotoxin level, sterility, storage stability and batch-to-batch consistency must be controlled under scalable GMP-compatible conditions. Highly complex multifunctional platforms may be difficult to commercialize if each component introduces additional variability in composition, potency, degradation and immune effect (263).

For this reason, clinically oriented TNBC nanomedicine should prioritize simplified, reproducible and modular designs. Platforms with clear mechanisms of action, validated potency assays, stable manufacturing processes and compatibility with standard chemotherapy or immunotherapy regimens are more likely to advance beyond proof-of-concept studies (264, 265).

### 4.4 Emerging biomimetic carriers and AI-assisted nanomedicine design

Biomimetic nanoparticles, extracellular vesicles and engineered immune-cell-derived carriers represent rapidly developing approaches for TNBC (266). Tumor-cell membrane coating may support homotypic targeting, platelet or macrophage membrane coating may recognize inflammatory or metastatic niches, and immune-cell-derived vesicles may carry immunomodulatory signals (267). However, their clinical use requires standardized cell sources, reproducible membrane extraction, cargo-loading methods, potency assays and safety testing (268).

Artificial intelligence and machine learning may further support nanomedicine development by predicting structure-property relationships, optimizing formulation parameters, modeling protein corona formation, estimating biodistribution, identifying patient-specific biomarkers and predicting toxicity or therapeutic response (269). Nevertheless, AI-assisted design must be validated experimentally and should not replace mechanistic biological evaluation (270).

### 4.5 Multi-omics-guided precision nanotherapy

**Figure 4:**
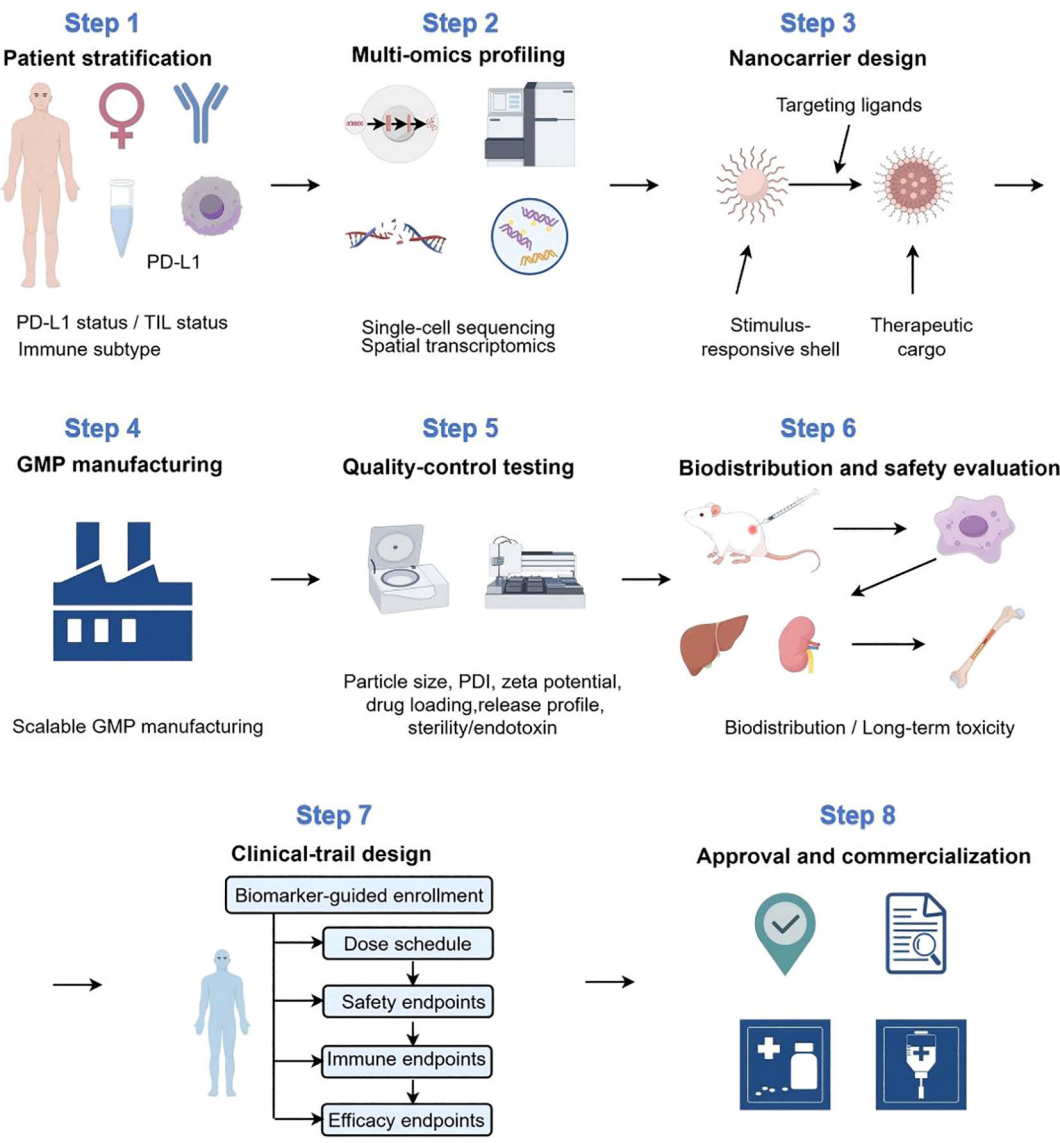
Proposed translational roadmap for TNBC nano-immunotherapy.

Single-cell sequencing, spatial transcriptomics, spatial proteomics, multiplexed imaging and patient-specific immune profiling can help identify which TNBC lesions are inflamed, immune-excluded, myeloid-dominant, stromal-rich or metabolically suppressive (**Figure 4**) (271). These data can guide the selection of targeting ligands, therapeutic cargos, release triggers and combination partners. For example, EGFR/TfR1/folate receptor expression may inform active targeting, PD-L1 and TIL status may guide ICB combinations, myeloid abundance may support TAM- or MDSC-directed strategies, and CAF/ECM-rich architecture may require stromal modulation before immune activation (272).

In this framework, TNBC nanotherapy should evolve from a one-size-fits-all delivery approach toward biomarker-matched and dynamically monitored precision therapy. Longitudinal sampling, liquid biopsy, imaging of nanoparticle deposition and immune profiling before and after treatment may allow adaptive selection of nanocarrier platforms and combination regimens (273).

## 5 Conclusion

In summary, nanodrug carriers have provided new research perspectives and therapeutic strategies for regulating the TIME in TNBC. Compared with traditional drug delivery approaches, nanocarriers can not only improve drug stability and enhance accumulation at tumor sites, but, more importantly, can also target key components such as TAMs, DCs, and T cells to regulate immune-cell polarization, enhance antigen presentation, induce immunogenic cell death, and reverse the immunosuppressive state. In selected preclinical or biomarker-defined settings, these strategies may promote immune activation, partially convert immune-cold phenotypes toward more inflamed states, and increase sensitivity to immunotherapy. Although this field still faces challenges such as heterogeneity, delivery efficiency, safety, and clinical translation, the continued development of materials science, tumor immunology, and precision medicine is expected to make intelligent nanocarriers for precise TIME regulation an important direction in the comprehensive treatment of TNBC and offer new possibilities for improving patient prognosis. Future progress will depend not only on simplified carrier design and clinically compatible materials, but also on the validation of practical stratification criteria that match specific nanocarrier functions with the immune, molecular, stromal, and vascular characteristics of individual TNBC lesions.
